# A Case of Necrotic Colonic Volvulus in Cerebral Palsy With Severe Scoliosis

**DOI:** 10.7759/cureus.56743

**Published:** 2024-03-22

**Authors:** Abdullah Alhelal, Ali M Assiri, Anas A Alqarni, Abdulrazak Tamim, Yazeed M Mohammad

**Affiliations:** 1 Pediatric Surgery, Abha Maternity and Children Hospital, Abha, SAU; 2 Pediatric Surgery, Saudi Ministry of Health, Abha, SAU; 3 Medicine and Surgery, College of Medicine, University of Bisha, Bisha, SAU; 4 General Surgery, Aseer Central Hospital, Abha, SAU

**Keywords:** pediatrics, surgical case reports, scoliosis, cerebral palsy, colonic volvulus

## Abstract

Cerebral palsy (CP) is a neurodevelopmental disorder that affects motor function and is often accompanied by secondary musculoskeletal issues. Severe scoliosis, a lateral curvature of the spine over 40 degrees, poses a significant challenge for individuals with CP, impacting their mobility and overall well-being. While the association between scoliosis and gastrointestinal complications is acknowledged, the occurrence of colonic volvulus with necrosis in the context of CP and severe scoliosis is rare and complex. This case report emphasizes the importance of clinical awareness in managing gastrointestinal complications in patients with CP and severe scoliosis.

An 11-year-old female presented with gastroenteritis and a concurrent viral upper respiratory tract infection. She experienced complications such as greenish vomiting, hematemesis, abdominal distention, and constipation. The patient has a medical history of epilepsy and was diagnosed with quadriplegic CP at four months old due to viral meningitis. She is currently on anti-epileptic medications and receives regular follow-ups with neurology. Severe lumbar scoliosis of more than 50 degrees Cobb angle is also noted. Physical examination revealed dehydration, bilious content in nasogastric tube (NGT) aspiration, tender abdomen, and an empty digital rectal examination. Some laboratory findings showed elevated levels of erythrocyte sedimentation rate (ESR), prothrombin time (PT), blood urea nitrogen (BUN), and sodium, while albumin levels were decreased, and white blood cell (WBC) count was mildly elevated. Abdominal computed tomography (CT) with contrast showed a distended ascending colon with air and swirling of the mesentery. The distal half of the large bowel was not dilated, and fecal matter was present. The small bowel appeared to be collapsed, and there was moderate free fluid in the peritoneal cavity, indicating colonic volvulus involving the proximal large bowel. The patient underwent surgery, which involved deflating and removing the distended colon, resecting the gangrenous colon, and performing an ilio-sigmoid anastomosis to restore gastrointestinal continuity. Postoperatively, the patient received close monitoring in the pediatric intensive care unit (PICU), received total parenteral nutrition (TPN) for five days, gradually progressed feeding, and showed overall improvement in her condition.

In conclusion, this case report highlights a rare occurrence of colonic volvulus in a patient with CP and severe scoliosis. It emphasizes the complex relationship between neurological and musculoskeletal disorders in gastrointestinal complications. A multidisciplinary approach is important for optimal management. It shows the importance of musculoskeletal factors in patients with neurological conditions. Overall, it contributes to the medical literature and emphasizes tailored management strategies for gastrointestinal issues in such patients.

## Introduction

Cerebral palsy (CP) is a neurodevelopmental disorder characterized by impaired motor function, often accompanied by secondary musculoskeletal complications [1]. Among these complications, severe scoliosis poses significant challenges, impacting not only mobility but also the overall health of affected individuals. While the association between scoliosis and gastrointestinal complications is acknowledged, the occurrence of colonic volvulus with necrosis in the context of CP and severe scoliosis is a rare and clinically intricate phenomenon [2].

Colonic volvulus, a condition where the bowel twists upon itself, can lead to severe consequences, including ischemia and necrosis [3]. The combination of CP and severe scoliosis adds a layer of complexity, as the altered anatomy and compromised motor function create a unique set of circumstances for gastrointestinal disorders such, as difficulty passing stools or infrequent bowel movements [4].

This case report aims to shed light on a distinctive clinical scenario involving a patient with CP and severe scoliosis who developed necrotic colonic volvulus. The rarity of such a case emphasizes the importance of recognizing and understanding the intricate interplay between neurological and musculoskeletal disorders such as dystonia, loss of selective motor control, and contractures in CP patients contributing to gastrointestinal complications [5].

Severe scoliosis, characterized by a lateral curvature of the spine exceeding 40 degrees, is known to have profound implications on respiratory and cardiovascular function. However, the association between severe scoliosis and gastrointestinal complications, particularly colonic volvulus, remains a less explored clinical entity [6].

The etiological factors leading to colonic volvulus in the presence of severe scoliosis are multifaceted. Colonic volvulus is linked to an enlarged colon and abdominal adhesions affecting mainly the sigmoid colon in adults, while in children majorly acting on the ileum. Furthermore, the neurologic impairment characteristic of CP could exacerbate the risk by affecting the autonomic control of bowel function.

Early diagnosis and intervention are paramount in mitigating the severe consequences of colonic volvulus. However, the complexity of this case underscores the importance of a multidisciplinary approach involving neurologists, orthopedic surgeons, and gastroenterologists to tailor a comprehensive management strategy.

Through this case report, we aim to contribute to the existing medical literature by presenting a rare and challenging case that highlights the need for heightened clinical awareness in managing gastrointestinal complications in patients with CP and severe scoliosis. Understanding the nuances of this complex interplay is crucial for optimizing patient outcomes and guiding future research into preventive and therapeutic strategies for similar cases.

## Case presentation

An 11-year-old Saudi female presented with a picture of gastroenteritis concomitant with a viral upper respiratory tract infection, CP, severe lumbar scoliosis, and epilepsy. Her condition was complicated by greenish vomiting and hematemesis with abdominal distention and then constipation. The patient is epileptic and diagnosed with CP at the age of 4 months post infancy viral meningitis. She is on anti-epileptic medications: Phenobarbital 50mg once daily, Topamax 25mg two times a day, Keppra 500mg two times a day, and Vagus nerve stimulation. On regular follow-up with neurology. She is known to have severe lumber scoliosis. On physical examination, the patient was found dehydrated. Greenish content was found in NGT aspiration. The abdomen was tender with an empty PR. Vitally stable except for tachycardia. The laboratory findings showed several abnormalities in the patient's test results. Specifically, the erythrocyte sedimentation rate (ESR), prothrombin time (PT), blood urea nitrogen (BUN), and sodium levels were elevated. However, other tests yielded results within normal ranges, except for a decreased level of albumin and a mildly elevated white blood cell (WBC) count (Table 1).

**Table 1:** Laboratory findings.

Test	Result	Normal Range
ESR (mm/hour)	49	0-15
PT (seconds)	29.8	11.5-15
APTT (seconds)	33.5	26-40
BUN (mmol/L)	34.5	6-24
Magnesium (mg/dL)	2.2	1.9-2.5
Potassium (mmol/L)	3.9	3.5-5.1
Sodium (mEq/L)	151.3	136-146
Calcium (mg/dL)	7.8	8.8-10.6
Phosphorus (mg/dL)	3.9	2.5-4.5
Albumin (g/dL)	2.7	3.5-5.2
WBC (10^9^/L)	10.08	4-10
Hb (g/dL)	13	12-15

**Figure 1:**
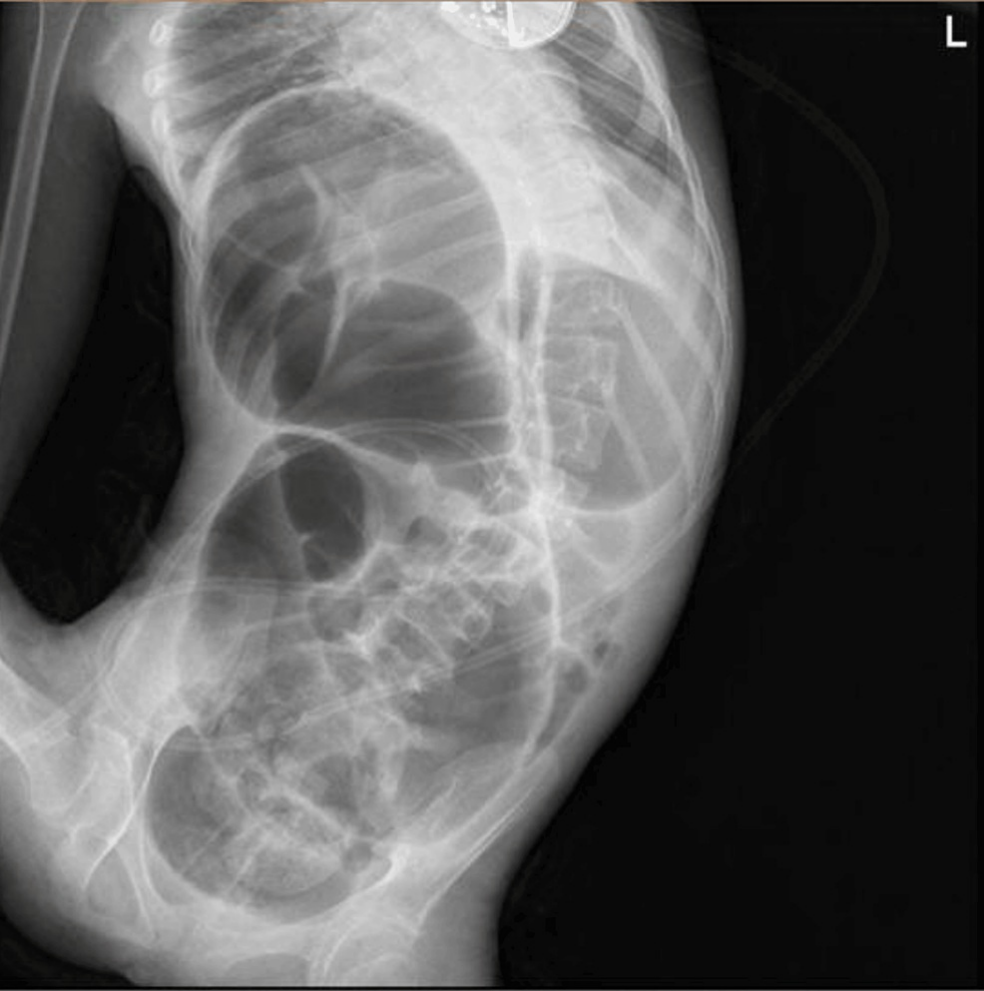
Frontal abdominal pre-operative x-ray. This figure shows diffuse Bowel Dilation: Extensive distension of bowel loops, indicative of an obstructive process, marked dilation of the bowel occupying almost the entire abdomen, potentially compressing other organs as well as elevating the right hemidiaphragm. Coffee Bean Sign: A loop of the bowel with a configuration reminiscent of a coffee bean, in keeping with volvulus. Lastly, it demonstrates lateral curvature of the spine, indicating scoliosis.

**Figure 2:**
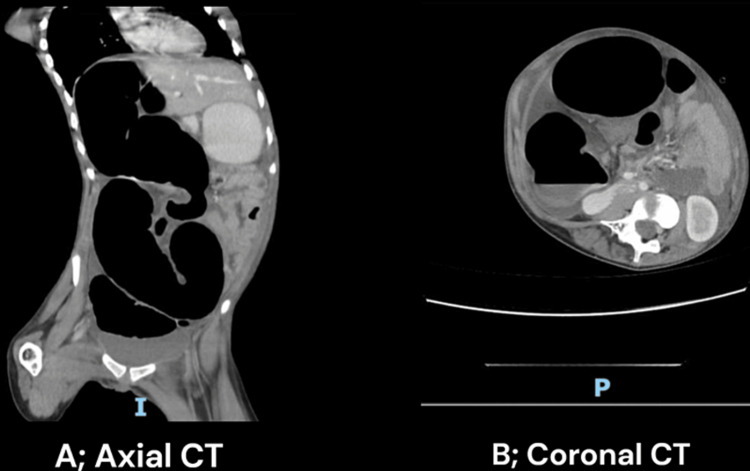
Axial (A) and coronal (B) sections of abdominal CT. It shows dilated obstructed proximal colon and collapsed descending colon.

Chest and abdominal x-rays were done (Figure 1). A computed tomography (CT) scan was performed using oral and intravenous contrast, which revealed a distended ascending colon with a maximum diameter of 8.5 cm. The colon contained predominantly air and exhibited some swirling of the mesentery. The distal half of the large bowel did not show any dilation and contained fecal matter. The small bowel appeared collapsed and not dilated. Additionally, there was a moderate amount of free fluid detected in the peritoneal cavity (Figures 2A, 2B). These findings indicated the presence of colonic volvulus involving the proximal large bowel, characterized by air distention.

**Figure 3:**
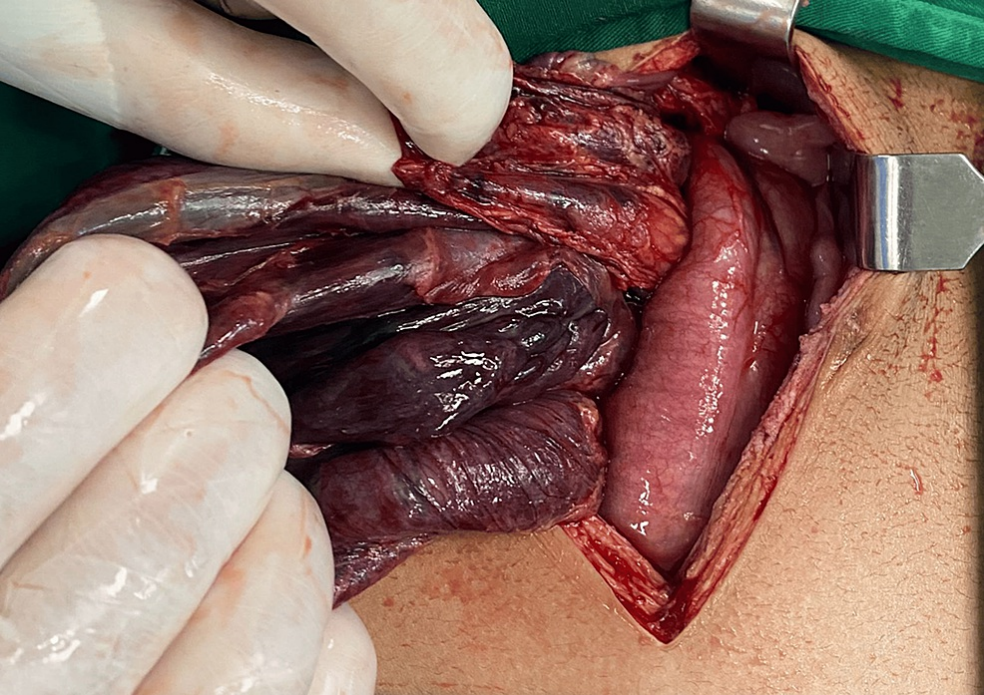
Necrotic bowel. This figure shows the state of the large bowel after it was deflated.

The treatment plan was to keep the patient NPO, NGT aspiration every three hours, and fleet enema TID for 24 hours. The patient was shifted to the operating room, where a midline laparotomy was done. During the operation, the presence of turbid bloody ascites was observed, in addition to the presence of cloudy fluid mixed with blood in the abdominal cavity. It was noted that the colon was distended and had a blackened, necrotized wall. Furthermore, the colon contained bloody content, as shown in Figure 3.

**Figure 4:**
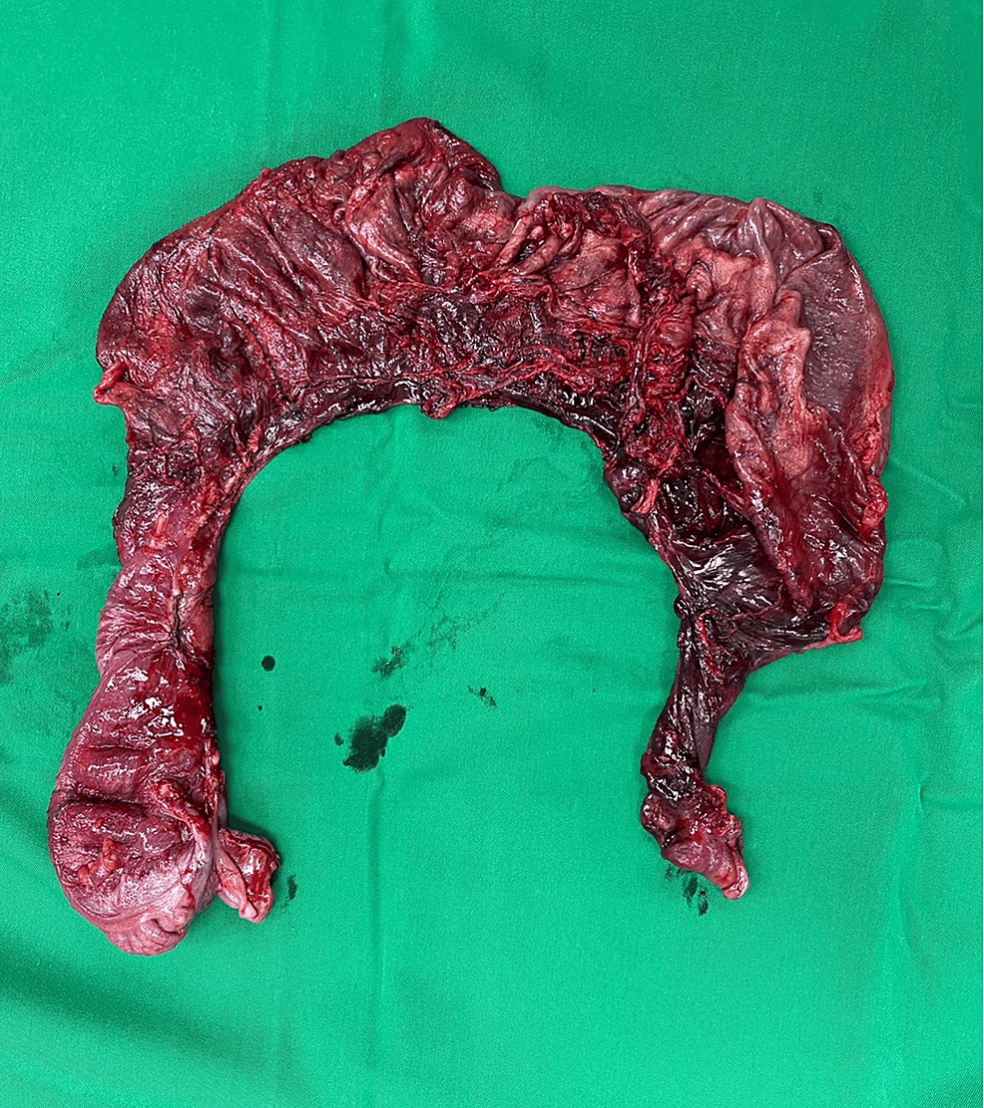
Resected gangrenous bowel. This figure shows the resected parts of the large colon, from the cecum up to descending colon.

To address the condition, the colon was deflated through aspiration trans the laparotomy wound. Approximately 2 liters of air and turbid bloody content were successfully removed from the colon. After deflating the colon, it was carefully delivered outside the abdomen. The surgical team identified a complete colonic volvulus involving the cecum, ascending colon, transverse colon, and descending colon. To treat the condition, a resection of the gangrenous colon was performed. Subsequently, an ilio-sigmoid anastomosis was carried out to restore the continuity of the gastrointestinal tract (Figure 4).

**Figure 5:**
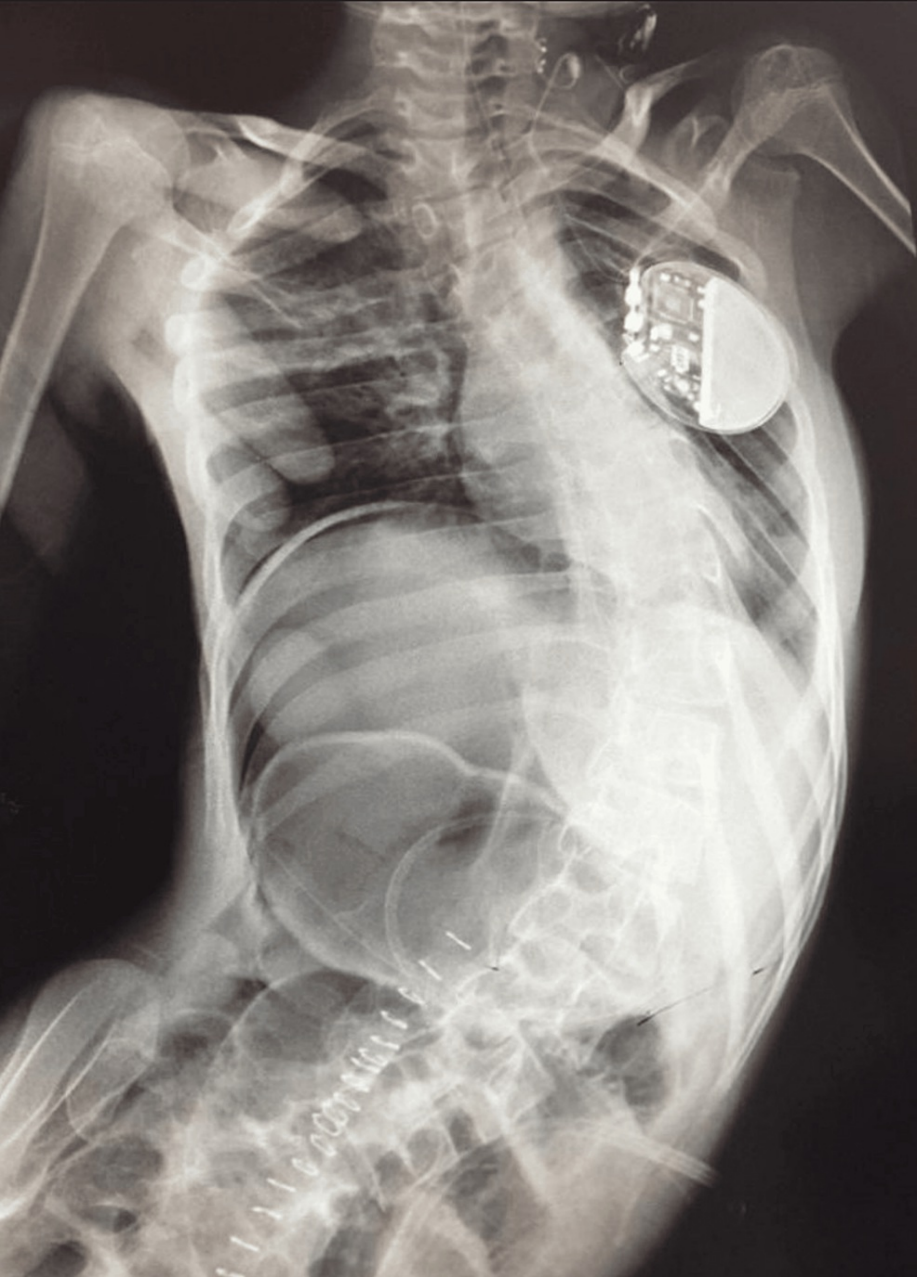
Post-operative x-ray. The bowel loops are less distended, which correlates with the expected postoperative changes following the resection of the volvulus. There is no obvious evidence of residual bowel obstruction. The right hemidiaphragm position appears normal.

Following the surgery, x-ray abdomen was repeated and shown in Figure 5. The patient was placed under postoperative care in the Pediatric Intensive Care Unit (PICU), where she received close monitoring. Total Parenteral Nutrition (TPN) was administered to the patient for a duration of five days to ensure proper nutritional support during the initial recovery phase. After the TPN period, the patient's diet gradually progressed, and she was started on progressive feeds. The feeding regimen was carefully adjusted until the patient reached the maximum feeds tolerated. Throughout this process, the patient showed overall improvement in her condition.

## Discussion

The presented case describes a complex clinical scenario involving an 11-year-old female with CP, severe lumbar scoliosis, and a rare complication of colonic volvulus. The unique combination of neurological impairment, musculoskeletal deformity, and gastrointestinal complications highlights the need for a comprehensive understanding of the interplay between these factors [7].

Individuals with CP often navigate a spectrum of challenges related to motor coordination and associated complications. The interplay between CP and colonic volvulus introduces a unique set of considerations [8]. Neurological impairment, as seen in CP, can influence colonic motility, potentially predisposing patients to gastrointestinal complications [9]. In this case, the patient's history of CP, diagnosed at the age of 4 months post-infancy viral meningitis, becomes a crucial element in understanding the pathophysiological factors contributing to colonic volvulus.

The compromised motor function in CP cases, may disrupt normal bowel movements, leading to alterations in colonic motility. Such abnormalities, when coupled with other factors such as severe scoliosis, can create an environment conducive to volvulus [10]. The rarity of reported cases addressing this specific association emphasizes the importance of recognizing gastrointestinal complications in the context of CP.

The diagnostic journey, encompassing chest and abdominal x-rays and a CT scan, played a pivotal role in identifying the extent of colonic involvement. The imaging studies indicated an elongated colon with distention in specific segments, providing insights into the structural factors that may have contributed to the development of colonic volvulus.

This patient’s presentation with greenish vomiting, hematemesis, and subsequent imaging revealing colonic volvulus serves as an important example of the intricate relationship between CP and gastrointestinal pathology. It sheds light on the need for heightened clinical awareness and tailored management strategies when dealing with colonic volvulus in individuals with CP [11,12].

The presence of severe lumbar scoliosis in conjunction with an elongated colon introduces an additional layer of complexity to the case. Scoliosis, characterized by lateral curvature of the spine, can lead to alterations in abdominal anatomy [13]. In this patient, the severe scoliosis likely contributed to an elongated and distorted colonic configuration, potentially creating a predisposition to volvulus [14].

The correlation between severe scoliosis and an elongated colon as potential risk factors for colonic volvulus underscores the importance of a holistic understanding of the patient’s anatomy. This case prompts a reflection on the need for careful evaluation of musculoskeletal factors in patients with neurological conditions, as these factors can significantly impact gastrointestinal dynamics and contribute to rare complications such as colonic volvulus [15].

The rarity of such cases emphasizes the importance of recognizing the diverse manifestations of gastrointestinal complications in this unique demographic. In a study by Hägglund et al., including pediatric patients with CP and severe scoliosis [16], may reveal an underreported incidence of colonic volvulus, underlining the need for increased clinical vigilance.

The patient's surgical intervention, involving colonic resection and ilio-sigmoid anastomosis, aligns with established protocols for managing colonic volvulus with necrosis [17]. This approach aims to address the ischemic segment of the colon, restore gastrointestinal continuity, and prevent further complications. The observed laboratory abnormalities, including elevated ESR, PT, BUN, and sodium levels, indicate the systemic impact of colonic volvulus on the patient’s overall health [18]. These markers, in conjunction with imaging findings, played a crucial role in guiding the diagnosis and subsequent management. Systemic manifestations of colonic volvulus in pediatric patients [13,19], could provide further insights into the prognostic significance of these laboratory parameters.

The postoperative care, including the administration of TPN and a gradual transition to enteral feeds, aligns with established practices in pediatric surgical recovery. A prospective study by Eveleens et al. evaluating nutritional support strategies in pediatric patients undergoing gastrointestinal surgeries could provide additional context for the chosen nutritional management in our case [20].

## Conclusions

In conclusion, this case report highlights the rare occurrence of colonic volvulus in a patient with CP and severe scoliosis, emphasizing the intricate interplay between neurological and musculoskeletal disorders in contributing to gastrointestinal complications. The presented case underscores the need for heightened clinical awareness and a multidisciplinary approach involving neurologists, orthopedic surgeons, and gastroenterologists in managing such complex scenarios. Understanding the nuances of this interplay is crucial for optimizing patient outcomes and guiding future research into preventive and therapeutic strategies for similar cases. Moreover, this case serves as a reminder to carefully evaluate musculoskeletal factors in patients with neurological conditions, as they can significantly impact gastrointestinal dynamics and contribute to rare complications. By shedding light on this distinctive clinical scenario, this case report contributes to the existing medical literature and emphasizes the importance of tailored management strategies for gastrointestinal complications in patients with CP and severe scoliosis.
